# Comparing self–other distinction across motor, cognitive and affective domains

**DOI:** 10.1098/rsos.240662

**Published:** 2024-10-09

**Authors:** Ekaterina Pronizius, Henryk Bukowski, Claus Lamm

**Affiliations:** ^1^ Department of Cognition, Emotion, and Methods in Psychology, Faculty of Psychology, University of Vienna, Vienna, Austria; ^2^ Faculty of Psychology and Educational Sciences, Psychological Sciences Research Institute, University of Louvain, Louvain-la-Neuve, Belgium

**Keywords:** self–other distinction, cognitive control, visual perspective-taking, automatic imitation, emotional egocentricity bias

## Abstract

The self–other distinction (SOD) is a process by which humans disentangle self from other-related mental representations. This online study investigated two unresolved questions: (i) whether partially the same processes underpin SOD for motor, cognitive and affective representations, and (ii) whether SOD overlaps with domain-general cognitive control processes. Participants (*N* = 243) performed three SOD tasks (motor: automatic imitation inhibition (AIT); cognitive: visual perspective-taking (VPT); affective: emotional egocentricity bias (av-EEB) tasks) and two cognitive control tasks (Stroop and stop-signal reaction time (SSRT) tasks). Correlation analyses showed no associations among the motor, cognitive and affective SOD indexes. Similarly, distinct SOD clusters emerged in the hierarchical clustering dendrogram, indicating clear separations among SODs. However, the results of multidimensional scaling suggested a tendency towards two clusters, as evidenced by the proximity of AIT and VPT indexes in relation to EEB indexes. AIT spatial laterality and Stroop domain-general cognitive control confounded AIT and VPT indexes, albeit slightly differently depending on the analysis method used. SSRT showed neither associations with SODs nor with other domain-general indexes. These findings underscore the complexity of SOD processes and have notable implications for basic and applied research, e.g. in the domain of clinical disorders affected by deficiencies in SOD.

## 1. Introduction

Social cognition comprises multiple processes that enable human beings to successfully navigate the social world [1]. During an interaction, individuals are prone to share the mental state of people around them, allowing them to sense the state the others are currently in (for reviews: [2–4]). For example, observation of someone in pain reactivates in a person neural structures involved in first-hand pain [5,6]. This sharing of representations related to the self and others occurs not only in the domain of emotions but also in the motor (for review: [7,8]) and cognitive domains (i.e. non-affective mental states; for review: 9). Parallel to this self–other sharing, humans possess a complementary process, the self–other distinction (SOD), to disentangle the self from other-related representations [2,3,9]. Without this ability, an excessive resonance with others, i.e. the emotional domain, would result in increased personal distress and reduced empathic concern towards others [4]. The present study aims to advance the current understanding of SOD by addressing two debated research questions.

*Research question 1: Regardless of the number or nature of the processes underpinning SOD, are these partially the same processes for motor, cognitive and affective mental representations?*

Three strands of SOD research have investigated three types of mental representations: motor, cognitive and affective representations. Motor SOD research is led by the finding that observing an action (motor content) induces a tendency to spontaneously imitate that action (for review: [7]). Motor SOD supports the inhibition of these imitative tendencies [10]. Cognitive SOD research is dominated by investigating processes related to a mental switch between self and other persons’ perspectives (e.g. how individuals represent other persons’ viewpoints; for review: [3,11]). Affective SOD research often relates to empathy research, where SOD allows the empathizer to recognize that their own affective state has been caused or influenced by another person’s affective state (for review: [2,12]).

The experimental measures of motor, cognitive and affective SOD have in common to contrast the performance between congruent and incongruent conditions. In the congruent condition, self- and other-related mental representations match (e.g. both experiencing positive emotion). In the incongruent condition, self- and other-related representation states are conflicting or opposite (e.g. the self experiences negative emotion, while the other experiences positive emotion). Neuroimaging studies found involvement of the temporoparietal junction (TPJ) for motor, cognitive and affective SOD, albeit with some localization differences (10; but see [13–15]; for review: [11,16]). So far, similarities in theories, operationalizations and neural correlates of motor, cognitive and affective SOD suggest similar processes underpinning SOD for the three types of mental representations. Yet, the few studies which addressed this question reported inconsistent findings [17–22].

The inconsistent findings raise at least three possible scenarios: (i) many of the previous studies may have been underpowered, (ii) the measurement tools of the SOD were built so that they fail to capture the underlying shared processes, (iii) the processes underlying SOD in motor, cognitive and affective domains may appear functionally similar but are not underpinned by the same (e.g. neural) processes.

For example, related to the second scenario, different neural activations found across the tasks might stem from input or other contextual differences and do not necessarily rule out the existence of shared processes across the three domains. The answer to research question 1 is thus debated, but there is little systematic research on this topic.

*Research question 2: To what extent do domain-general processes confound SOD(s)?*

Another intriguing question is what role domain-general processes, which help individuals overcome similar computational challenges in non-social situations, play in SOD. In other words, is SOD achieved solely through processes specialized for social cognition, or do domain-general processes also contribute, and to what extent, in addressing these challenges?

Similar computational challenges result from the fact that in both kinds of situations, individuals have to detect and resist an interference. A prominent example from a non-social domain is the Stroop task [23], in which participants are instructed to react to the words’ colour and not words’ lexical meaning. In the incongruent trials, the colour and the meaning do not correspond, causing interference that requires conflict detection and inhibition. Same domain-general cognitive control processes might account for conflict detection and inhibition in the social domain, in which participants detect and resist an interference between their mental state and the mental state of another social agent.

The question of domain-generality is particularly debated in cognitive SOD with level-1 visual perspective-taking (VPT) tasks (level-1 refers to inferring whether someone sees an object or not). Specifically, it is still debated whether VPT performance necessarily involves inferring an actual mental state [24] or if it is the product of a domain-general mechanism named submentalizing (25–31; for review: [32,33]; meta-analysis: [34]). Studies have empirically supported both positions, leading to contradictory results (but see [35–37] for an intermediary position).

The debate also exists in research on motor SOD. There, it was shown that generalized visuospatial effects may confound automatic imitation [38,39]. Spatial confounding occurs when changes in task performance are influenced by the task-irrelevant match (or mismatch) between the laterality of the expected response and the laterality of the stimulus [40]. Recent meta-analyses, including fMRI studies [13,41], supported the generalist view, according to which this paradigm involves general processes to a greater extent than aspects specialized for social cognition. At last, SOD and tasks of the cognitive control contrasting incongruent to congruent conditions typically activate the TPJ, with however some socio-cognitive versus cognitive control differences [42,43].

Hence, similarities in theories, operationalizations and neural correlates of SOD and cognitive control raise the debate about whether and to what extent domain-general processes support the SOD(s) [32,33,44]. Our second research question is phrased more broadly than question 1. We foresee at least two different scenarios: (i) any shared processes identified in Q1, if they exist, are used for wider cognitive control processes; alternatively, (ii) each SOD may rely to a different extent on domain-general processes.

### 1.1. The present study

The aim of the present study was, first, to identify whether processes underpinning motor, cognitive and affective SOD overlap, at least in part. Second, whether SOD(s) is, to a larger extent, a process involved in interference resolution used for broader cognitive, social and non-social computational challenges. An early theoretical paper that emphasized the potential advantage of considering SOD across the three domains comes from Eddy [4]. Our study is the first to empirically investigate these two questions through multiple *correlation analyses, hierarchical clustering* and *multidimensional scaling*. To this end, we concurrently examined performance-based measures of motor, cognitive and affective SOD and domain-generality in a well-powered within-subject experimental design.

For this purpose, we used three well-established SOD tasks (motor SOD: [45]; cognitive SOD: [46]; affective SOD: [47]), and two control tasks, targeting individual differences in domain-general cognitive control (Stroop task, [23]; Stop stimulus reaction time (SSRT) task, [48,49]). For the motor representations, we used a task version, which explicitly disentangles general spatial congruency from automatic imitation [45].

## 2. Methods

### 2.1. Data collection

We ran a pilot test between September and October 2020. The data collection took place between November and December 2020.

### 2.2. Sample

The study sample was recruited via The Vienna CogSciHub: Study Participant Platform of the University of Vienna (https://cognitivescience.univie.ac.at/services/study-participant-platform/). Potential participants, recruited via the *hroot* recruitment tool [50], received the link to the online platform of the study, where they underwent a screening process by filling out a short questionnaire. The exclusion criteria were: (a) age under 18; (b) left-handedness according to the Edinburgh Handedness Inventory (EHI [51]); (c) colour blindness; (d) hearing impairment; (e) psychological, psychiatric or neurological disorders; (f) substance abuse; and (g) poor German skills. Participants received either partial monetary compensation based on 10 euros per hour if the study was not completed or 30 euros for completing all tasks. The study was conducted according to the Declaration of Helsinki (1964) and its later amendments and was approved by the Ethical Board of the University of Vienna, Faculty of Psychology (EK reference number 00577, amendment 5 to project 00412).

Three hundred and fifty-eight (*n* = 358, see §2.5.1 for power considerations) participants registered in the study, out of which *n* = 310 finished at least one task. In each task, we excluded participants who either made too many errors (>20%—a criterion determined before the analyses and without peeking at the data, though not preregistered), experienced technical difficulties, violated the model’s assumption, or explicitly stated in writing at the end of the task that they did not understand the instructions or were not concentrated. The final sample comprised *n* = 243 individuals who completed all five tasks. The majority of the sample self-identified as women (*n* = 198), had university entrance qualifications (*n* = 158; *n* = 59 completed their bachelor’s degree) and were of Austrian nationality (*n* = 135; *n* = 72 Germany). The sample mean age was 23.47 (s.d. = 5.17, age range: 18–64).

### 2.3. Tasks

The online study consisted of the three SOD tasks, two control tasks and one survey, presented in a randomized order.

The classification of tasks into different domains of SOD (motor, cognitive and affective) is based on the mental state being interfered with (motor intention, person-centred viewpoint and emotional state, respectively), the type of stimuli used to evoke these mental states, and the associated neural correlates. We based our definition of these domains on conventions established in previous research (e.g. [7,14,15]).

With regards to the control tasks, the Stroop task is a classic measure of inhibitory cognitive control and has a longstanding tradition of being used in the context of automatic imitation inhibition [7,20,52]. We chose a version with manual responses, though there has been some debate about whether different response modes (manual versus vocal) produce similar effects [53,54]. Our choice for the manual response mode was motivated by a better alignment with the cognitive and motor demands of other RT paradigms in our study (e.g. in terms of task complexity and response mode, such as pressing or lifting a key).

The SSRT task measures the ability to inhibit a prepotent response, i.e. how quickly an individual can inhibit an initiated action. The SSRT (or variations of it) has been often used as a control task in the context of VPT [19]. To prevent overburdening our participants with additional cognitive control tasks like change detection [55] or set-shifting [56] and to ensure our results are well-contextualized within the existing literature, we selected these two tasks as our control measures.

The practice trials, attentional and technical checks and further additional details for every task can be found in electronic supplementary materials [57].

#### 2.3.1. Automatic imitation inhibition task

The automatic imitation inhibition task (AIT) measures the SOD processes for motor mental representations [10,45,58]. We used a version proposed by Sowden & Catmur [45] to isolate imitative congruency from the confounded effects of spatial congruency.

**Figure 1.**
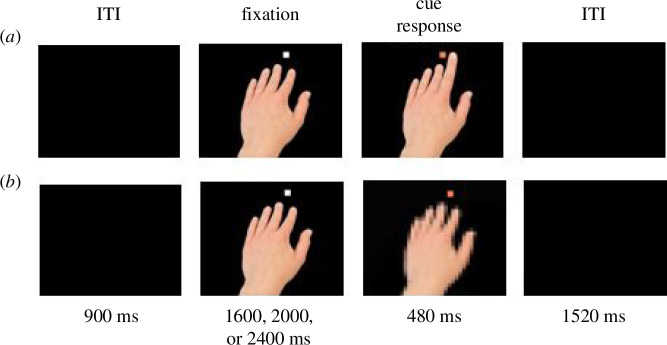
Timeline of (*a*) an Experimental Trial; (*b*) a Base Trial. Note: A trial starts with an intertrial interval, followed by a frame depicting a static hand alongside a white fixation square. In the experimental trials (*a*), the next frame shows a lifted finger. In the base trials (*b*), the stimulus hand remained static and was additionally pixelated. In this case, an orange fixation square provides a cue for a finger response (group 1: middle finger, group 2: index finger lift). The trial ends with a 1520 ms inter-stimulus interval. The next trial starts only when both N and M keys are pressed. Adapted from [45, fig. 1]; Open Access article distributed under the terms of the Creative Commons Attribution Non-Commercial License.

The participants placed the index and middle fingers of their right hand on the keyboard (index = *N*, middle = *M*). During the trials, they were instructed to lift their fingers in response to a coloured cue (group 1: purple = index finger lift, orange = middle finger lift; group 2: vice versa). This cue appeared on display together with the task-irrelevant right or left stimulus hand of a female person lifting the fingers. The stimulus hand was executing the same (imitative congruent) or opposite (imitative incongruent) finger movements (figure 1). Viewing the hand’s movements activates the participants’ involuntary imitation tendencies, which need to be inhibited actively in the incongruent trials [10]. This results in prolonged reaction times and more errors compared with congruent trials, which represent a quasi-imitative reaction [10]. Spatial congruency was manipulated in an orthogonal manner to imitative congruency, depending on whether the stimulus hand lifted a finger that was on the same (spatial congruent) or on an opposite (spatial incongruent) side of space as the finger to be lifted by the participant.

The experimental part comprised three experimental blocks with 36 trials per block. Within each block, four experimental conditions and two base conditions, with six trials per condition, were distributed in a pseudorandomized manner. The stimulus hand movements were manipulated in a 2 (congruency: congruent versus incongruent) × 2 (spatial versus imitative) fashion, resulting in four experimental trial types. In the base trials, the stimulus hand (right or left) did not perform any movement. The task took approximately 15 minutes to complete.

#### 2.3.2. VPT task

**Figure 2.**
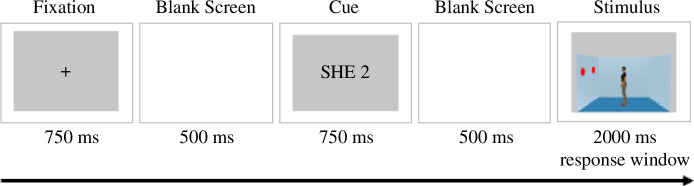
Timeline of an experimental congruent trial. Note: The trial starts with a fixation cross, followed by an inter-stimulus interval (ISI). The next frame indicates to the participants that they have to adopt another person’s perspective (she) and memorize the digit 2. After the second ISI, the participant sees a gender-matched avatar (e.g. a woman) facing the wall with the two red discs on it. The correct response is yes since the memorized digit 2 matches the number of discs the avatar can see. In this example, the participant and the avatar see the same number of discs (congruent condition). Adapted from [46], stimuli: Samson & Apperly, 2015, Figshare [59].

The VPT task proposed by Samson *et al.* [46] measures the SOD processes for cognitive mental representations. The participants placed two fingers on the *C* and *N* keys of their keyboard. In every trial, participants adopted either self or other perspective and memorized a digit (0–3) that appeared on the screen. The next frame depicted a lateral view of a room. In the centre of this room stood a gender-matched human avatar facing either the right or the left wall. Simultaneously, red discs were displayed on either one or two walls. The participants were instructed to verify if the memorized digit matches the number of the discs the agent (self or other) can see (yes = *C*, no = *N*) (figure 2).

The stimuli were manipulated in a 2 × 2 × 2 fashion (perspective: self versus other, congruency: congruent versus incongruent, match: matching versus mismatching). The matching trials were always associated with a ‘yes’ response, and the mismatching trials with a ‘no’ response. In the congruent condition, the avatar and the participant always saw the same number of discs. In the incongruent condition, some of the discs were not visible to the avatar but were visible to the participant. It was shown that participants could not easily ignore the irrelevant perspective [46], resulting in prolonged reaction times and more errors in the incongruent condition compared with the congruent condition. Depending on the cue prompted to take the self-perspective or the avatar’s perspective, the interference on performance due to the conflict of viewpoints is referred to as the altercentric bias or egocentric bias, respectively.

In the main experimental part, there were four blocks, each consisting of 48 trials, along with four filler trials and no feedback. These trials encompassed 24 self trials and 24 other trials, with an equal variation of congruency and match trial type. The trials were presented in a pseudorandom order with no more than three consecutive trials of the same type. In the filler trials, no discs were shown on the walls. The task took approximately 22 min to complete.

#### 2.3.3. Audiovisual emotional egocentricity bias task

The audiovisual version of the emotional egocentricity bias task (av-EEB) developed by von Mohr *et al*. [47] measures the SOD processes for affective mental representations. The tasks consisted of five parts: deception, stimuli familiarization, catch trials, manipulation check (described in electronic supplementary material, 3) and the main experimental part.

In the main experimental part, the participants were falsely led to believe that they performed the online task together with another person. Each trial involved participants hearing a sound and viewing a picture on the screen. The picture represented the type of sound the fake participant was supposedly exposed to. Participants adopted either the self or other perspective depending on the block they were in, and in each trial, they rated the pleasantness of the audiovisual stimulation from the adopted perspective.

**Figure 3.**
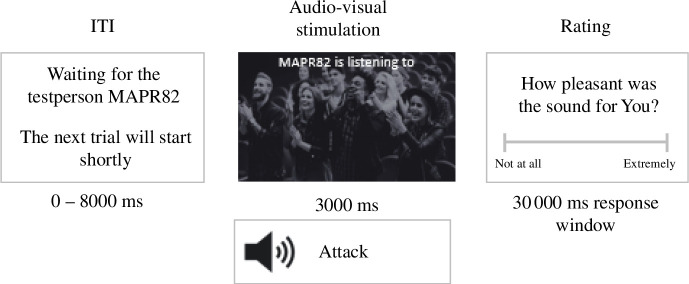
Timeline of an experimental incongruent self-trial. Note. A trial starts with an intertrial interval, followed by a picture depicting an applauding crowd. The text on the top of the picture indicates that the paired fake participant MAPR82 is listening to sounds associated with this picture (in this case, of a positive valence). The real participant is listening to the sound of a woman being attacked (negative valence), making it an incongruent trial. After 3000 ms, the participant had to rate their feelings induced by the attack audio stimulation (self-rating). Based on the study by [47], adapted from https://psyarxiv.com/j7vec/, CC0 1.0 Universal.

For the self-perspective, the participants rated the pleasantness of the sound they heard. For the other perspective, the participants had to provide an estimation of how pleasant the sound depicted as the picture was for the other person (figure 3). The stimuli were manipulated in a 2 × 2 × 2 fashion (perspective: self versus other, congruency: congruent versus incongruent, valence: positive versus negative).

Each trial was classified as either pleasant or unpleasant based on the valence of the stimulation directed at the target (self or other). For example, if the target was ‘other’ and the stimulation was unpleasant (e.g. a picture of a dentist with a drill), while the participant heard a sound of a positive valence (e.g. a laughing baby), then the trial was classified as ‘other unpleasant’. Detailed information on the stimuli valence can be found in electronic supplementary material, 3.

Congruent trials involved audiovisual stimulation of the same valence for both perspectives, while incongruent trials had the self and the other experiencing audiovisual stimulation of opposite valences. In this type of setup, incongruent trials are typically associated with reduced ratings (‘shifted’ towards the valence of the participant’s stimulation [15,47]) for the target compared with congruent trials.

The main experimental part consisted of four blocks (two self and two other blocks) with 16 trials per block presented in a randomized order. From these 16 trials, eight were of pleasant valence (four congruent and four incongruent trials), and eight were of unpleasant valence (four congruent and four incongruent trials). For the ratings, we deployed a visual analogue scale ranging from −10 (not at all) to +10 (extremely). The task took approximately 30 min to complete.

#### 2.3.4. Stroop task

The Stroop task [23] measures the general ability to inhibit prepotent response tendencies (a part of cognitive control). The participants placed their fingers on the predefined keys (left middle = *C*, left index = *V*, right index = *N*, right middle = *M*). During the trials, they were instructed to press a key in response to the colour of a presented stimulus (group 1: red = *C*, green = *V*, blue = *N*, yellow = *M*; group 2: red = *N*, green = *M*, blue = *C*, yellow = *V*). The presented stimuli were either colour words (RED, GREEN, BLUE, YELLOW) or four X letters (XXXX). In the congruent trials, the colour words were written in a matching colour (e.g. BLUE printed in blue colour). In the incongruent trials, the colour of the words did not match their lexical meaning (e.g. BLUE printed in red colour). Therefore, in the incongruent trials, the participants had to suppress or actively inhibit a highly automatic response tendency (reading a word), resulting in prolonged reaction times and more errors than in congruent trials [23]. During the baseline trials, the four X letters appeared in one of the four possible colours (either red, green, blue or yellow).

The participants were instructed to react as fast and as accurately as possible. In the main experimental part, four blocks with 30 randomized trials per block and no feedback were presented (see electronic supplementary material, 4 for a trial timeline). Out of these 120 trials, 40 were congruent trials, 40 were incongruent trials and 40 were baseline trials. The task took approximately 12 min to complete.

#### 2.3.5. SSRT task

The SSRT is a paradigm to measure the inhibition of prepotent response tendencies (similar to the Stroop task). The participants respond with a button press to certain stimuli (go trial) while withholding a response to other stimuli (stop trial). Our task implementation procedure followed the recommendations provided by Verbruggen *et al*. [49]. The participants placed their fingers on the *V* and the *N* keys. During the go trials, they were instructed to press a key in response to a direction indicated by an arrow (*V*—when the arrow stimulus was pointing to the left, *N*—to the right). In the stop trials, in which the participants had to inhibit their response tendencies, the arrow turned red after a variable delay period (stop-signal delay (SSD), see electronic supplementary material, 5 for a trial timeline).

The participants were instructed to react as fast and as accurately as possible. In addition, they were explicitly encouraged not to wait for a stop signal. The SSD was continuously adapted to an individual reaction time so that a successful inhibition would have been possible in 50% of the stop trials, and the number of go omissions would have been close to 0%. In the main experimental part (after the practice block), four blocks with 64 randomized trials per block were used, and no feedback was presented. Out of these 64 trials, 48 were go trials and 16 were stop trials. The task took approximately 20 min to complete.

### 2.4. Indexes

#### 2.4.1. The balanced integration scores in AIT, VPT and Stroop tasks

To control for a possible speed-accuracy trade-off in the AIT, VPT and Stroop tasks, we combined per condition the reaction times and accuracy (proportion correct) into the ‘Balanced Integration Score’ (BIS) using the function BIS ([60]; https://github.com/Liesefeld/BIS). The BIS is designed to measure how well a participant balances accuracy and speed. It is calculated by subtracting the standardized reaction time from the standardized percentage correct for each observation per condition. Higher BIS indicate efficient performance where participants manage to achieve high accuracy with relatively low reaction times. Conversely, lower BIS suggests a potential trade-off, where either speed or accuracy is compromised.

#### 2.4.2. Automatic imitation inhibition task

Prior to index calculation, we discarded trials that deviated ± 2.5 s.d. from the participant’s mean RT or were faster than 150 ms. Then, we combined per condition the reaction times and accuracy (proportion correct) into the BIS.

First, we computed the spatial congruency index by subtracting the BIS scores in spatial congruent trials from the corresponding spatial incongruent trials (equation (2.1)).


AIT spatial congruency bis index=

(2.1) $$\frac{\left({\text{SIIC}}^{\text{MeanBIS}}+{\text{SIII}}^{\text{MeanBIS}}\right)}{2}-\frac{\left({\text{SCIC}}^{\text{MeanBIS}}+{\text{SCII}}^{\text{MeanBIS}}\right)}{2},\;$$

where BIS = balanced integration score; SC = spatial congruent; SI = spatial incongruent; IC = imitative congruent, II = imitative incongruent (e.g. SCII—spatial congruent, imitative incongruent trial).

Our data showed (see https://osf.io/nys7q/, *Task Validation*) that spatial incongruence had a stronger interference effect than imitative incongruence. As a result, when struggling to resist the interference due to more dominant spatial incongruence, the participants might have had limited processing resources left to withstand the imitative incongruency. Consequently, and in contrast to the original study, we further refined the task’s indexes by cancelling out the more dominant spatial incongruency. Specifically, we computed the subsequent imitative indexes based solely on spatially congruent trials, aiming to enhance comparability with other SOD measures without a double perceptual conflict.

We computed the imitative congruency index on spatial congruent trials (equation (2.2)) by subtracting the BIS scores in imitative congruent trials from the imitative incongruent trials. Negative difference suggests that incongruent trials are associated with poorer performance (either slower reaction times or lower accuracy, or both). A positive difference indicates less interference and better SOD.


(2.2) $$\text{AIT imitative congruency sp bis index}\;={\text{SCII}}^{\text{MeanBIS}}-{\text{SCIC}}^{\text{MeanBIS}},\;$$

where BIS = balanced integration score; sp = index calculated on spatial congruent trials; SC = spatial congruent; IC = imitative congruent; II = imitative incongruent.

In line with Bukowski, Todorova *et al*. [17], we calculated the inhibition costs index on spatial congruent trials (equation (2.3)). The inhibition costs is the adjusted SOD score.


(2.3) $$\text{AIT inhibition costs}\;sp\;bis=\left(\frac{{\text{BR}}^{\text{MeanBIS}}+{\text{BL}}^{\text{MeanBIS}}}{2}\right)-{\text{SCII}}^{\text{MeanBIS}},\;$$

where BIS = balanced integration score; sp = index calculated on spatial congruent trials; BR = base right hand; BL = base left hand; SC = spatial congruent; II = imitative incongruent.

#### 2.4.3 VPT task

As in the original study by Samson *et al.* [46], we excluded mismatching trials (‘no’ responses, see electronic supplementary material, 2, *n* = 24 trials per block). Next, we discarded trials that deviated ±2.5 s.d. from the participant’s mean RT or were faster than 150 ms. Finally, we combined per condition the reaction times and accuracy (proportion correct) into the BIS score.

First, we computed two biases by comparing the BIS scores in the incongruent trials to the congruent trials for the two perspectives equations (2.4) and (2.5). Negative difference suggests that incongruent trials are associated with poorer performance. Positive difference indicates less interference and better SOD.


(2.4) $$\text{VPT Altercentric bis bias}={\text{Incongruent Self}}^{\text{MeanBIS}}-{\text{Congruent Self}}^{\text{MeanBIS}},\;$$

(2.5) $$\text{VPT Egocentric bis bias}={\text{Incongruent Other}}^{\text{MeanBIS}}-{\text{Congruent Other}}^{\text{MeanBIS}},\;$$

where BIS = balanced integration score.

The altercentric bias describes a human’s tendency to be influenced by the inferred mental state of others when building judgements about one’s own mental state [36]. In other situations, individuals sometimes egocentrically build their judgements on their own experience when trying to infer the mental states of those around them [15,36].

As in AIT, we calculated the adjusted SOD score (VPT conflict index, equation (2.6)). VPT conflict is the main effect of incongruency and corresponds to the mean of altercentric and egocentric biases.


(2.6) $$\begin{array}{r} \text{VPT Conflict bis}=\left(\frac{\left({\text{Incongruent Self}}^{\text{MeanBIS}}+{\text{Incongruent Other}}^{\text{MeanBIS}}\right)}{2}\right)\\ \;\;\;-\left(\frac{\left({\text{Congruent Self}}^{\text{MeanBIS}}+{\text{Congruent Other}}^{\text{MeanBIS}}\right)}{2}\right),\; \end{array}$$

where BIS = balanced integration score.

#### 2.4.4 Audiovisual emotional egocentricity bias task

In the baseline rating analysis, we aimed to replicate the design of the original study [47]. Our results showed stimuli fit for all but one item (electronic supplementary material, 3.). The audio and picture stimuli of bees were rated neutral and not negative as intended. This motivated us to exclude all trials from the main analysis where the bees stimuli were used (25% of all trials).

We calculated several indexes quantifying the ability to correctly distinguish between self and other perspectives, making it comparable with the VPT task [17,46]. The altercentric bias is calculated by comparing the ratings in the incongruent trials to the congruent trials in the self condition (equation (2.7)). The egocentric bias is calculated by comparing the ratings in the incongruent trials to the congruent trials in the other condition (equation (2.8)).


(2.7) $$\text{EEB }\text{Altercentric }\text{bias}={\text{Incongruent Self}}^{\text{MeanR}}-{\text{Congruent Self}}^{\text{MeanR}},\;$$

(2.8) $$\text{EEB Egocentric bias}={\text{Incongruent Other}}^{\text{MeanR}}-{\text{Congruent Other}}^{\text{MeanR}},\;$$

where as per condition, we aggregated the mean judgements for pleasant and unpleasant stimuli as follows: [pleasant judgements + (−1) × unpleasant judgements]/2. *R* = Rating.

Note that in both the AIT and VPT tasks, researchers, including ourselves, have observed that reaction times are typically slower for incongruent trials compared with congruent trials. Transforming these reaction times into BIS scores reverses this pattern: we would expect higher BIS scores for congruent trials compared with incongruent trials. The negative difference between incongruent and congruent (incongruent – congruent) trials suggests that incongruent trials are associated with poorer performance and greater interference.

In the EEB task, incongruent trials are associated with lower (i.e. less extreme) ratings compared with congruent trials [15,47]. In practical terms, this means that when calculating interference as the difference between incongruent and congruent trials (incongruent – congruent), a lower (more negative) EEB index value consistently reflects greater interference, aligning with the methodology used in the other two tasks. This consistency allows for a more straightforward comparison of results across different experimental conditions.

As in AIT and VPT tasks, we calculated the adjusted SOD score (EEB conflict index, equation (2.9)). The EEB conflict index is the main effect of incongruency and corresponds to the mean of altercentric and egocentric biases.


(2.9) $$\begin{array}{r} \text{EEB Conflict}=\left(\frac{\left({\text{Incongruent Self}}^{\text{MeanR}}+{\text{Incongruent Other}}^{\text{MeanR}}\right)}{2}\right)\\ -\left(\frac{\left({\text{Congruent Self}}^{\text{MeanR}}+{\text{Congruent Other}}^{\text{MeanR}}\right)}{2}\right),\; \end{array}$$

where as per condition, we aggregated the mean judgements for pleasant and unpleasant stimuli as follows: [pleasant judgements + (−1) × unpleasant judgements]/2. *R* = Rating.

#### 2.4.5. Stroop task

Prior to index calculation, we discarded trials that deviated ± 2.5 s.d. from the participant’s mean RT or were faster than 150 ms. Next, we combined per condition the reaction times and accuracy (proportion correct) into the BIS scores.

The Stroop congruency index was computed by subtracting the BIS scores in congruent trials from the incongruent trials (equation (2.10)). Negative difference suggests that incongruent trials are associated with poorer performance. Positive difference indicates less interference.


(2.10) $$\text{Stroop congruency bis}={\text{Incongruent}}^{\text{MeanBIS}}-{\text{Congruent}}^{\text{MeanBIS}},\;$$

where BIS = balanced integration score.

Next, we computed the inhibition costs index (equation (2.11)).


(2.11) $$\text{Stroop inhibition costs bis}={\text{Baseline}}^{\text{MeanBIS}}-{\text{Incongruent}}^{\text{MeanBIS}}.\;$$

Note that, due to how the indexes are constructed, the Stroop congruency index closely resembles the altercentric indexes of SOD, while the Stroop inhibition costs index aligns more closely with the conflict indexes of SOD. This aspect has been taken into account when specifying the relevant models for the domain-generality research question.

#### 2.4.6. SSRT task

We estimated the SSRT index using the integration method, which, according to Verbruggen *et al*. [49], is less biased and more reliable than other methods (e.g. the mean method). The index was calculated with the R Package SSRTcalc (integration_adaptiveSSD function [61]). Adaptive refers to an adaptive method of setting SSD, as in our case an increase/decrease by 50 ms, depending on whether participants successfully inhibited their prepotent response tendencies in the stop trials or not. In the integration method (equation (2.12)), SSRT is computed by subtracting the mean SSD from the reaction time on the nth trial, where n is the number of RTs in the RT distribution of go trials multiplied by *p*(respond|signal). Higher values indicate a reduced ability to inhibit prepotent tendencies.

To make it comparable with other tasks, where higher values indicate less interference, we reversed the SSRT score by multiplying it by (−1).


(2.12) $${\text{SSRT}}_{\text{integration}}=\left(-1\right)\times {\text{RT}}_{\text{nth}}-M_{\text{SSD}},\;$$

where RT = reaction time; *M* = mean; SSD = stop-signal delay.

### 2.5. Statistical analyses

#### 2.5.1. Power consideration

*Correlation analysis:* To ensure stable estimates, the sample size should ideally approach *n* = 250 [62,63]. Acknowledging the challenges posed by sample recruitment during the COVID−19 pandemic, our minimum targeted sample size was set at *n* = 150, following Qureshi *et al*. [19], while the maximum *N* was capped at 300, consistent with Shaw *et al.* [64]. Similar to ours in the methodology, these studies investigate the structure of social cognition.

#### 2.5.2. Correlation analyses

The analyses were run on the final sample of the participants who completed all five tasks (*n* = 243). All indices were standardized before the analyses to reduce the influence of possible outliers.

Related to our main question 1, ‘*Regardless of the number or nature of the processes underpinning SOD, are these partially the same processes for motor, cognitive and affective mental representations?*’, we predicted that should the corresponding indexes of SOD performance for motor, cognitive and affective mental representations measure the same underlying processes, they will significantly correlate with each other.

To investigate this assumption, we clustered indexes into three meaningful groups of indexes:

1. Altercentric indexes: AIT imitative index calculated on spatial congruent trials and controlled for possible speed-accuracy trade-offs (sp BIS), VPT altercentric bias (BIS) and EEB altercentric bias;
2. Egocentric indexes: VPT egocentric bias (BIS) and EEB egocentric bias.
3. Conflict indexes: AIT inhibition costs (sp BIS), VPT conflict index (BIS) and EEB conflict index.

Regarding our second main question, ‘*To what extent do domain-general processes confound SOD(s)?*’, we predicted that should the corresponding indexes of SOD performance for motor, cognitive and affective mental representations measure domain-general processes, they will significantly correlate with the indexes of domain-general cognitive control performance.

To investigate the domain-general SOD hypothesis, we added the cognitive control indexes to the most fitted groups. In the case of the Stroop task, we calculated indexes using a similar approach as in the SOD tasks. As a result, the Stroop congruency index (BIS) fitted best to the altercentric group and the Stroop inhibition costs (BIS) to the conflict group. However, we did not have specific methodological or theoretical justifications regarding the AIT spatial (BIS) and SSRT index classification. Therefore, we added them to all model groups, that is the altercentric, egocentric and conflict groups and computed the respective correlations.

As a reminder, the spatial congruency index of the AIT task captures the influence on performance caused by whether the laterality of the expected response and laterality of the stimulus match or not [45,65]; the Stroop congruency index measures the interference on performance caused by the stimuli conflict between reading and colour perception [23]; the SSRT index quantifies the interference on performance caused by the conflict between expected response and an irrelevant response [48,49].

For every research question, we adjusted the significance alpha level to control for multiple comparisons. Given the three theoretical clusters (altercentric, egocentric and conflict), the adjusted *α* = 0.017.

#### 2.5.3. Hierarchical clustering and multidimensional scaling

Hierarchical clustering and multidimensional scaling are exploratory data analysis methods to identify and visualize patterns and groupings within the data. In the present paper, these two methods have been applied to the scaled data to reduce the influence of potential outliers.

Hierarchical clustering proceeds successively by merging smaller clusters into larger ones [66]. The results of this process can be visualized in a dendrogram—a tree of clusters—which shows how the clusters are related [66]. Longer branches represent a greater dissimilarity, while shorter branches represent a greater similarity between the clusters. Clusters that merge earlier (at lower levels) are more similar to each other than clusters that merge later (at higher levels).

To analyse these relationships between the variables, we first calculated the pairwise Euclidean distances between the scaled observations, by applying an *R* function *dist*. Next, we performed hierarchical clustering using the average linkage method based on the distance matrix with the R function *hclust* [67].

Multidimensional scaling is used to visualize similarities or dissimilarities in data by representing them in a two-dimensional space [68]. Variables or data points that are more similar in the original dataset are depicted as closer to each other in this space. Conversely, dissimilar variables or data points are positioned farther apart [68]. To analyse these relationships between the variables, we first calculated the pairwise Euclidean distances between the scaled observations, by applying an *R* function *dist*. Next, we performed multidimensional scaling with the R function *cmdscale* [67].

### 2.6. Open science statement/transparency and openness

#### 2.6.1. Open data and code

The data and the data analysis scripts for all experiments are available at https://osf.io/nys7q/ [doi: 10.17605/OSF.IO/NYS7Q]. The study was not preregistered.

#### 2.6.2. Study materials

The stimuli used in the tasks are not openly shared, as they have been obtained from the authors of the original studies upon request.

#### 2.6.3. Additional questionnaires (not part of the present paper)

All participants completed a set of questionnaires measuring traits and states in different domains of social, cognitive and emotional functioning: (i) Behavioural Inhibition and Behavioural Activation System scale (BIS/BAS) [69], a scale measuring sensitivity to punishment (BAS) and sensitivity to reward and approach motivation (BAS); (ii) Connectedness with the most closest person or community; (iii) Positive and Negative Affect Scale [70], a scale measuring subjective experience of positive and negative affect; (iv) Patient Health Questionnaire 9 (PHQ−9) scale, a diagnostic instrument for assessing depression severity [71]; (v) Generalized Anxiety Disorder−7 (GAD−7) scale, a diagnostic instrument for assessing severity of anxiety [72]; (vi) Questionnaire of cognitive and affective empathy (QCAE) scale, an instrument for measuring trait empathy [73]; (vii) Toronto Alexithymia Scale (TAS), an instrument for measuring difficulties in understanding, processing and describing emotions [74]; (viii) UCLA Loneliness Scale (UCLA−3), a self-report measure of loneliness [75]. The results of these questionnaires are not part of the present study and will be reported elsewhere.

#### 2.6.4. Tasks validation

Prior to index calculation, we conducted task validation analysis. On the project’s OSF page [https://osf.io/nys7q/], readers can find the task validation code and the final written report. In this written report and the present manuscript, we follow Loenneker *et al*. [76] to transparently document and justify our RT preprocessing decisions. Here, we omitted the §2.6.4 to enhance the manuscript’s conciseness, thereby improving readability and maintaining clarity for the readers.

Briefly, the results for all tasks replicated expected findings based on prior studies, with the notable exception of the AIT task. In the AIT task, we uncovered a new pattern of results in terms of a significant interaction between spatial and imitative congruencies as a function of the sample size. Notably, the AIT task developed by Sowden & Catmur [45] was tested for the first time on a large sample and was specially designed to disentangle spatial from imitative congruencies.

#### 2.6.5. Software

Data were analysed using R, version 4.3.0 [67] and the R packages: *apaTables* [77,78], *dendextend* [79], *dplyr* [80], *effectsize* [81], *emmeans* [82], *flextable* [83], *ggplot2* [84], *ggpubr* [85], *here* [86], *interactions* [87], *jtools* [88], *lmerTest* [89], *mergeutils* [90], *officer* [91], *rio* [92], *Rmisc* [93], *sjPlot* [94], *SSRTcalc* [61], *tibble* [95], *tidyr* [96], *tidyverse* [97] and *writexl* [98].

## 3. Results

The descriptive results are reported in table 1.

**Table 1.** Indexes mean and s.d. (prior scaling).

no	variable	mean	SD	no	variable	mean	SD
1	AIT imitative sp bis	0.03	1.21	7	EEB altercentric	−0.76	1.24
2	AIT inhibitioncosts sp bis	−0.02	1.14	8	EEB egocentric	−1.17	1.73
3	AIT spatial bis	0.00	1.17	9	EEB conflict	−0.96	1.19
4	VPT altercentric bis	−0.04	1.16	10	Stroop congruency bis	−0.12	1.21
5	VPT egocentric bis	0.05	1.35	11	Stroop inhibitioncosts bis	0.05	1.30
6	VPT conflict bis	0.00	0.92	12	SSRT	−226.23	40.88

Note. *n* = 243. AIT = automatic imitation inhibition task, VPT = visual perspective-taking task, EEB = audiovisual-visual emotional egocentricity bias task, SSRT = stop-signal reaction time task. sp = indexes calculated on spatial congruent trials; bis = indexes controlled for speed-accuracy trade-off. AIT, VPT and Stroop bis composite scores: Negative values suggest that incongruent trials are associated with poorer performance, while positive values indicate less interference and better SOD. EEB scores: A lower (more negative) EEB index value reflects greater interference. SSRT Scores: The SSRT scores were reversed (multiplied by −1) to align the direction of interference with other tasks, with negative values now indicating greater interference.

### 3.1. Correlation analyses

Correlation analysis revealed no significant between-tasks correlations in the altercentric group of indexes (table 2).

**Table 2.** Correlation table. Altercentric bias.

variable	1	2	3	4	5
AIT imitative sp bis					

VPT altercentric bis	−0.12				
	[−0.24, 0.01]				

EEB altercentric	−0.08	−0.01			
	[−0.21, 0.04]	[−0.14, 0.12]			
	
Stroop congruency bis	0.12	−0.00	0.03		
	[−0.01, 0.24]	[−0.13, 0.12]	[−0.10, 0.15]		
		
AIT spatial bis	0.05	0.12	0.00	0.11	
	[−0.08, 0.17]	[−0.01, 0.24]	[−0.12, 0.13]	[−0.01, 0.24]	
			
SSRT	−0.01	−0.06	0.05	0.03	0.04
	[−0.13, 0.12]	[−0.18, 0.07]	[−0.07, 0.18]	[−0.10, 0.15]	[−0.09, 0.16]
				

Note. This table presents Pearson correlation coefficients with pairwise deletion calculated with the function *apa.cor.table* from the R Package *apaTables* [77,78]. Values in square brackets indicate the 95% confidence interval for each correlation. *n* = 243. AIT = automatic imitation inhibition task, VPT = visual perspective-taking task, EEB = audiovisual-visual emotional egocentricity bias task, SSRT = stop-signal reaction time task. sp = indexes calculated on spatial congruent trials; bis = indexes controlled for speed-accuracy trade-offs. *p < 0.05, **p < 0.01.

In the egocentric group, neither were the SOD between-tasks correlations significant, nor were the correlations between SOD indexes and the domain-general indexes (table 3).

**Table 3.** Correlation table. Egocentric bias.

variable	1	2	3
VPT egocentric bis			

EEB egocentric	0.05		
	[−0.08, 0.17]		

AIT spatial bis	0.06	−0.02	
	[−0.07, 0.19]	[−0.14, 0.11]	
	
SSRT	−0.01	−0.04	0.04
	[−0.13, 0.12]	[−0.16, 0.09]	[−0.09, 0.16]
		

Note. This table presents Pearson correlation coefficients with pairwise deletion calculated with the function *apa.cor.table* from the R Package *apaTables* [77,78]. Values in square brackets indicate the 95% confidence interval for each correlation. *n* = 243. AIT = automatic imitation inhibition task, VPT = visual perspective-taking task, EEB = audiovisual-visual emotional egocentricity bias task, SSRT = stop-signal reaction time task. sp = indexes calculated on spatial congruent trials; bis = indexes controlled for speed-accuracy trade-off. *p < 0.05, **p < 0.01.

Correlation analysis revealed no significant between-task correlations in the SOD inhibition costs group of indexes (table 4). AIT inhibition costs on spatial congruent trials controlled for speed-accuracy trade-off correlated with AIT spatial index (*r* = 0.54, *p* < 0.001).

**Table 4.** Correlation table. Inhibition costs.

variable	1	2	3	4	5
AIT inhibitioncosts sp bis					

VPT conflict bis	0.07				
	[−0.05, 0.20]				

EEB conflict	−0.04	−0.01			
	[−0.16, 0.09]	[−0.13, 0.12]			
	
Stroop inhibitioncosts bis	−0.03	0.04	0.02		
	[−0.15, 0.10]	[−0.09, 0.17]	[−0.10, 0.15]		
		
AIT spatial bis	0.54**	0.12	-0.01	0.02	
	[0.44, 0.62]	[−0.01, 0.24]	[−0.14, 0.11]	[−0.11, 0.14]	
			
SSRT	0.00	−0.04	−0.00	−0.04	0.04
	[−0.12, 0.13]	[−0.17, 0.08]	[−0.13, 0.12]	[−0.17, 0.09]	[−0.09, 0.16]
				

Note. This table presents Pearson correlation coefficients with pairwise deletion calculated with the function *apa.cor.table* from the R Package *apaTables* [77,78]. Values in square brackets indicate the 95% confidence interval for each correlation. *n* = 243. AIT = automatic imitation inhibition task, VPT = visual perspective-taking task, EEB = audiovisual-visual emotional egocentricity bias task, SSRT = stop-signal reaction time task. sp = indexes calculated on spatial congruent trials; bis = indexes controlled for speed-accuracy trade-off. *p < 0.05, **p < 0.01.

### 3.2. Hierarchical clustering and multidimensional scaling

The results of the hierarchical cluster analysis visualize the similarities and dissimilarities between the variables. The SSRT variable, being the most distant from all others, has been removed from the dendrogram to improve graph clarity.

**Figure 4.**
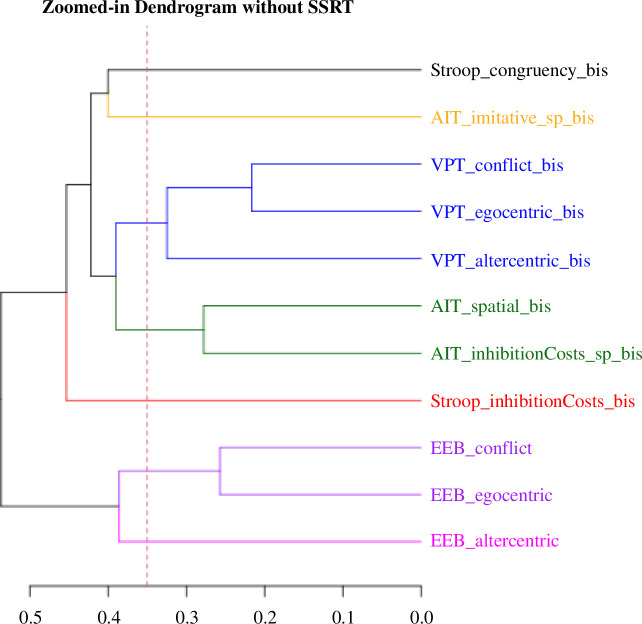
Results of the hierarchical cluster analysis. Note: The SSRT variable is not displayed on this graph due to its significant distance from other variables (53.99). Its exclusion is intentional to enhance graph clarity. In the provided dendrogram, a horizontal line is drawn at a height of 0.35. This cut-off was selected based on the largest vertical gaps between merges, indicating significant separations between clusters. By examining the dendrogram, it is evident that cutting at this height allows for the identification of distinct groups within the data.

Upon visually examining the dendrogram (figure 4), it becomes evident that all three SODs (motor, cognitive and affective) form at first distinct clusters. This is despite the performed preprocessing steps aimed at minimizing measurement-related ‘noise’, such as addressing speed-accuracy trade-offs in tasks like AIT and VPT, or additionally reducing spatial interference in the AIT indexes.

Notably, the VPT and EEB tasks exhibit a similar grouping pattern: the conflict index and egocentric bias are initially grouped together, followed by the altercentric tendencies. This implies that despite the conflict index representing the mean of egocentric and altercentric tendencies, it predominantly captures processing akin to egocentric tendencies.

Regarding domain-general processes, the Stroop congruency index was more closely related to the AIT imitative index, whereas spatial processes formed a separate cluster with the AIT inhibition costs. In a subsequent step, the Stroop inhibition costs index merged with the motor–cognitive cluster, entailing domain-general processes. And the EEB indexes were, if anything, minimally associated with the domain-general processes. Finally, in the very last step, all clusters were merged with the SSRT index (not shown in the figure).

**Figure 5.**
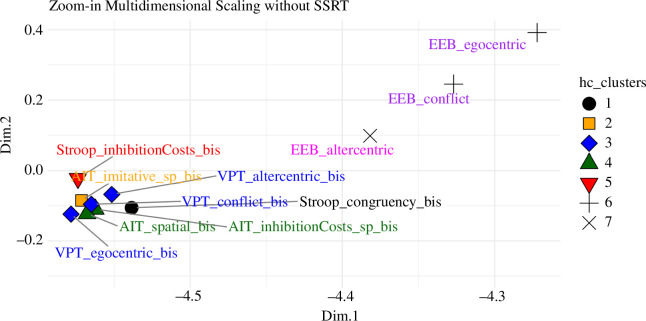
Results of the multidimensional scaling. Note: The SSRT score is not displayed on this graph due to its significant distance from other scores (Dim1: 49.49, Dim2: 0.004). Its exclusion is intentional to enhance graph clarity. hc_clusters = Clusters identified through hierarchical clustering analysis.

The results of multidimensional scaling (figure 5) mostly coincide with those of hierarchical clustering. Note that, to enhance clarity, the SSRT score, the most distant index, has been excluded from both graphs but not from the respective analyses.

Consistent with the results of the hierarchical clustering, the SSRT (not shown in the figure) is positioned the farthest from all other indexes. The EEB indexes are notably distant, and both Stroop indexes are somewhat closer to the motor–cognitive cluster. The motor–cognitive cluster itself includes the VPT and AIT indexes, along with the domain-general indexes of spatial processes.

Overall, the MDS analysis indicates a similarity between the Stroop, VPT and AIT indexes, whereas the EEB indexes (along with SSRT) stand out as being distinct.

## 4. Discussion

The present study addressed two main questions: (i) whether partially the same processes underpin the SOD for motor, cognitive and affective representations and (ii) whether the SOD can be explained by domain-general processes related to cognitive control and thus may not be specific to social cognition. For these purposes, we employed online versions of the three SOD tasks pervasively used in the literature (motor SOD: [45]; cognitive SOD: [46]; affective SOD: [47]) and two domain-general cognitive control tasks (Stroop task: [23]; SSRT: [48,49]). First, we discuss how our findings answer each question and then what additional insights our results convey regarding understanding of socio-cognitive processes.

*Research question 1: Regardless of the number or nature of the processes underpinning SOD, are these partially the same processes for motor, cognitive and affective mental representations?* The correlation analysis showed no significant between-task associations. Similarly, separate task clusters emerged during the hierarchical clustering. However, the multidimensional scaling placed the AIT and VPT indexes closer together than the EEB indexes. These results provide correlational evidence that the SOD may not involve similar or the same mechanisms consistently applied across motor, cognitive and affective mental representations. The MDS two-dimensionality aligns with neuroimaging studies, which have shown that the motor–cognitive SOD activates the same brain area (rTPJ), whereas the SOD for affective representations engages a neighbouring but distinct brain structure [3].

*Research question 2: To what extent do domain-general processes confound SOD(s)?* To investigate the extent to which the SOD is specific to the domain of social cognition, we additionally included indexes considered as measures of domain-general processes. First, our correlation analyses revealed that stimulus laterality (AIT spatial congruency) correlated with one of the AIT indexes, specifically the AIT inhibition costs. The results from multidimensional scaling and hierarchical clustering showed that AIT spatial congruency and the Stroop indexes were close to the motor–cognitive cluster, which comprised the AIT and VPT indexes. While these confounding effects have already been extensively discussed in the motor–cognitive domains [40,99,100], our study is among the few to specifically address the role of domain-general processes in the affective SOD. The results of the present study provide no correlational support for the involvement of these domain-general processes in affective SOD.

Finally, SSRT inhibition of prepotent responses has shown no relationship with either the SOD tasks or Stroop indexes, indirectly supporting previous findings that the Stroop task and the SSRT task involve different cognitive processes [101].

In summary, our correlative findings align with some previous literature favouring the involvement of domain-general processes in motor–cognitive SOD (for review [32, 38]). Yet, the lack of significant correlations between the SOD indexes and the domain-general indexes, combined with their relative distance in the dendrogram and multidimensional graph, suggests that SOD cannot be considered equivalent to a domain-general mechanism related to inhibitory control. Fundamentally, with our findings, we highlighted a very complex structure of SOD [19].

### 4.1. Study strength

As discussed in the §1, previous inconsistent findings raised at least three possible scenarios.

First, many of the previous studies were underpowered. The strength of the present study is a relatively large sample size, which is sufficient for correlation analysis to provide an effect close to the true effect, given (or assuming) low task reliability [62,102].

Second, it is possible that similarities or dissimilarities of the measurement tools could at least partially confound the results. For example, almost all tasks, except for the audiovisual EEB task, are reaction time task paradigms. Additionally, both the AIT and VPT tasks involve spatial compatibility and rely on body position in relation to the environment. To rule out this scenario, we implemented several strategies to cancel out measurement noise, such as accounting for both reaction time and accuracy (BIS scores; [102,103]) introducing an alternative index of SOD (‘conflict index’) to achieve a more accurate representation of the construct, and cancelling out the dominant spatial incongruence in the automatic imitation task. If the theoretical assumptions underlying data preprocessing hold true, then the resulting indexes are not confounded by measurement differences and should represent true underlying processes. Therefore, our results are consistent with the third scenario: the SOD processes for motor–cognitive and affective mental representations may appear functionally similar but are not underpinned by the same shared processes.

### 4.2. Limitations

An alternative explanation of our results might be that the failure to find associations across tasks might still be of a methodological and not conceptual nature, relating to, first, how reliably these tasks can capture individual differences [102]. The VPT task shows modest to good test–retest reliability [31] and has proven useful for detecting individual differences [19,64,104–106]. The automatic imitation task shows high split-half reliability [107], but to our knowledge, there has been no formal assessment of test–retest reliability. However, the AIT task robustly detects individual differences (meta-analysis: [41]), including the specific task version used in our study [58]. Less is known for the only recently introduced audiovisual EEB task; in its first use, a relationship was shown between interindividual differences in interoceptive accuracy and the EEB [108]. In another study, low test–retest reliability was suggested [109]. Note that this study introduced modifications to the task that render the results non-informative for the present study.

Secondly, the lack of association between the EEB task and the other SOD indexes or domain-general indexes might be attributed to the response differences (RT versus Ratings) and not to differences in the underlying mechanisms, despite our attempts to reduce measurement noise. The AIT, VPT and Stroop tasks are characterized by slower reactions in the incongruent trials compared with the congruent trials. Transforming the reaction times into scores reverses this pattern, making it so that higher BIS scores indicate better performance. In the EEB task, incongruent trials are characterized by less extreme ratings, indicating a shift towards the valence of the participant’s own stimulation. By transforming the reaction time tasks’ scores using the BIS function and calculating the contrasts between incongruent and congruent conditions across all tasks, we believe we have successfully aligned the direction of the interference. This transformation allows us to better compare the degree of interference across tasks by making the data more consistent. However, it is still possible that, despite our efforts, the two-dimensionality might reflect inherent task-related differences rather than a unified measure of interference.

Thirdly, for both the AIT and VPT tasks, the focus is on the external self, specifically body position in relation to the environment and other people. This is different from the EEB task, which requires affect-relevant (internal state) judgements for the self and others. However, by computing relative indices, some of these possible confounds were likely to be removed, as they were constant across the task conditions. While we are convinced that our data preprocessing cancels out differences in the self-focus and movements, isolating the handling of interference in its pure form, it is essential to recognize that current behavioural SOD tasks require improvement or replacement to advance the field.

Although we utilized the most advanced tools available to capture SOD, further developments in behavioural SOD tasks are necessary. That said, the tasks we used have a longstanding tradition of effectively measuring SOD. Consequently, we opted for the approach presented in this paper—assessing SOD across three domains using three well-validated tasks—rather than creating a new task with unknown and potentially less validated psychometric properties. In the latter scenario, a null effect of the present study would more likely be attributed to the novelty of the task (and its unknown properties) rather than the nature of SOD.

This highlights a significant limitation in the research on SOD mechanisms: the absence of a single task that can address multiple instances of SODs while controlling for confounding variables. An example of such a task from a higher order social domain is the EmpaToM, which assesses empathy and theory of mind using the same stimuli and within the same task structure [110]. Similarly, there is no dedicated SOD questionnaire, unlike the numerous questionnaires available for measuring empathy [e.g. 73]. Furthermore, a recent ecological momentary study on empathy found that different components of empathy often co-occur in daily life [111], making it challenging to investigate each facet, such as affective SOD, in isolation.

Certainly, SOD can be operationalized in various ways beyond the approach presented in this paper. For instance, cognitive SOD can be measured by assessing the ability to differentiate self and other-related beliefs or desires, rather than focusing solely on visual perspectives. Affective SOD can be assessed by evaluating automatic emotional disentanglement or by involving allocation of specific emotions (within a wide range, e.g. happy, angry, bored, etc.) to the self versus other. Exploring new ways of operationalizing SOD, along with addressing other limitations, represents promising avenues for future research. Yet, until a single task for the three domains or an SOD questionnaire is developed, the scientific community will need to continue relying on converging evidence from different empirical studies or studies utilizing various experimental paradigms within a single sample.

Lastly, the absence of correlations between Stroop and SSRT indexes may question the effectiveness of our analysis strategy in identifying a domain-general cognitive control process. Yet, our study intentionally aimed to cover distinct facets of cognitive control, avoiding an overly narrow focus on just one aspect. An additional question might arise about whether the control tasks used were an appropriate choice. Our results indicate that the Stroop task is closely related to the AIT and VPT tasks, suggesting that they share some similarities, making our choice appropriate. However, the SSRT task has been less informative, so it may be worth considering replacing it with other control tasks, such as those related to change detection [55] or set-shifting [56], in future replications.

#### 4.2.1 Constraints on generality

Following Simons *et al*. [112], our constraints on generality statement will help other researchers evaluate the scope and generalizability of our claims. We believe that the results of our study can be generalized to individuals with similar demographic characteristics in terms of age, gender and education.

Our convenience sample comprised 310 participants who performed at least one task. Only 243 of those completed all five tasks and were included in the final analysis. Given our focus on examining the effects across the five tasks, we implemented strict exclusion criteria, which meant only considering the data from those who completed every task with fewer than 20% errors, experienced no technical difficulties, or explicitly stated that they performed the task according to instructions (self-reported). Since data collection was done online, these strict exclusion criteria served to verify that participants performed the tasks according to our directives.

While this approach ensures that our analysis is based on a consistent and complete dataset, it introduces potential bias. Excluding participants who did not complete all tasks may inadvertently exclude individuals with certain traits relevant to social cognition, such as motivation, attention span or internet access stability, which might systematically differ between those who completed all tasks and those who did not. Additionally, excluding participants with more than 20% errors is common practice in RT research. Still, this practice ignores the fact that a large number of errors can represent a true underlying effect. Consequently, our findings might not fully represent the broader population, as the excluded participants could have provided valuable insights that are now missing from the analysis.

Importantly, our data collection took place during the acute phase of the COVID-19 pandemic, including social distancing and lockdowns. It remains unclear how this time of uncertainty influenced the participants’ underlying SOD ability and, as a consequence, their task performance. Furthermore, we tested social cognition and cognitive control with simple experimental paradigms. While these methods are well-suited to study underlying processes, it is yet to be determined how these findings translate to the outside real world.

### 4.3. Future directions

Our correlational study, although informative, cannot definitively eliminate the alternative explanation that the results are driven by the differences in the measurement tools utilized to assess the SOD. To further advance scientific understanding of SOD underlying processes, progress in measuring SOD is needed, such as a development of a single task with alternating motor, cognitive and affective representational contents or via alternative SOD measures capturing individuals’ everyday social life, using an ecological momentary approach.

Next, in contrast to correlation studies as the present study, interventional and neurostimulation studies are needed to provide causal evidence of the processes involved in SOD. However, targeting these specific processes presents methodological challenges both at the behavioural [17] and the brain [104] levels.

Further, one could argue that the results of our analyses depend upon many parameters chosen by the researcher (see multiverse debate 113, 114), which questions the replicability of the findings. We propose that the methodological choices made in our study set a new standard for future research. For example, unlike past research, we have excluded spatially incongruent trials in the AIT task, the *bees* trials in the EEB task, and transformed the RT indexes into BIS scores [60]. The version of the AIT task we used [45] is relatively new, and not much work has been done on it. We believe (and our data show) that, contrary to what the creators of the task expected, the interaction between spatial and imitative compatibilities will be an issue when the sample size is large. Therefore, we foresee that future studies will follow our approach to remove spatially incongruent data. Similar to the AIT task, the audiovisual version of the EEB task [47] is new and would benefit from a larger stimulus pool. Finally, using BIS scores [60] is a common procedure to control for speed-accuracy trade-offs. For readers interested in how our results fit within the context of past research, we advise referring to our *Task Validation* report and online data (https://osf.io/nys7q/), which were not included in the present manuscript to maintain conciseness.

Overall, in the present study, we adopted a combined approach of confirmatory and exploratory investigation [115]. While our intention was to confirm SOD one-dimensionality, we achieved this by transparently employing diverse exploratory methods, contributing to a broader and more extensive understanding of the cognitive processes involved. Validating the observed result pattern with new data [116] and integrating it with past and future research is crucial to ascertain the robustness and generalizability of the findings.

In light of this, it becomes imperative to consider the necessity for investigations within clinical populations to further elucidate the underlying mechanisms. Overall, our results represent an essential intermediate phase in the progression from basic to clinical research. For instance, Eddy [117] discussed the differences and similarities between schizophrenia and Tourette syndrome regarding social functioning. Both clinical populations showed increased internal simulations of others’ actions and emotions but reduced mentalizing [117]. However, some of the deficits might have been confounded by general cognitive impairment [117]. Given the heterogeneity of symptoms, specifically in schizophrenia, it is not surprising that the empirical results are often mixed. For example, Simonsen *et al*. [118] reported intact top-down modulation of imitation in patients with schizophrenia, whereas Rudolph *et al*. [119] found the opposite.

Our study can encourage psychiatric research to investigate SOD across at least three domains (motor, cognitive and affective) and to include measures of general cognitive abilities. If we assume domain independence, based on the results of the current correlation study, we might expect different disorders to exhibit disorder-specific impairments in one of the domains, alongside overall nonspecific impairments in general cognitive mechanisms. Therefore, clinical work could be particularly informative by identifying specific SOD impairments that contribute to symptoms in various psychiatric conditions. Another prediction is that impairments in one domain might be associated with enhanced SOD in another domain as a compensatory mechanism for disorder-specific deficits, potentially uncovering markers of resilience. Given the impairment of SOD in several psychiatric conditions, such as borderline personality disorder [120], SOD is viewed as a transdiagnostic mechanism [4] and, therefore, a strategic target for interventions.

## 5. Conclusion

In the present study, we addressed two highly debated questions: (i) whether partially the same processes underpin the SOD for motor, cognitive and affective representations and (ii) whether or to what extent the SOD involves domain-general processes related to cognitive control. For these purposes, we employed online versions of the three well-established SOD tasks and the two domain-general cognitive control tasks on a large sample (*n* = 243).

Our study not only provides an extensive investigation of SOD on multiple levels but also highlights the complex structure of SOD and reveals novel association patterns when considering conflict SOD index, controlling for spatial compatibility and accounting for speed-accuracy trade-offs. The converging results suggest a potential two-dimensionality between motor–cognitive and affective representational contents. Crucially, the general ability to inhibit prepotent response tendencies measured by the Stroop task and the interference from stimulus laterality seem to play a role in the motor–cognitive SOD.

## Data Availability

The data and the data analysis scripts for all experiments are available at [121]. The study was not preregistered. The stimuli used in the tasks are not openly shared, as they have been obtained from the authors of the original studies upon request. The current manuscript (including its previous forms) has been published as a preprint on OSF [122]. Supplementary material is available online [57].

## References

[1] Frith CD. 2008 Social cognition. Philos. Trans. R. Soc. Lond. B Biol. Sci. **363**, 2033–2039. (10.1098/rstb.2008.0005) 18292063 PMC2375957

[2] Lamm C, Bukowski H, Silani G. 2016 From shared to distinct self-other representations in empathy: evidence from neurotypical function and socio-cognitive disorders. Philos. Trans. R. Soc. Lond. B Biol. Sci. **371**, 20150083. (10.1098/rstb.2015.0083) 26644601 PMC4685528

[3] Steinbeis N. 2016 The role of self-other distinction in understanding others’ mental and emotional states: neurocognitive mechanisms in children and adults. Philos. Trans. R. Soc. Lond. B Biol. Sci. **371**, 20150074. (10.1098/rstb.2015.0074) 26644593 PMC4685520

[4] Eddy CM. 2022 The transdiagnostic relevance of self-other distinction to psychiatry spans emotional, cognitive and motor domains. Front. Psychiatry **13**, 797952. (10.3389/fpsyt.2022.797952) 35360118 PMC8960177

[5] Lamm C, Decety J, Singer T. 2011 Meta-analytic evidence for common and distinct neural networks associated with directly experienced pain and empathy for pain. Neuroimage **54**, 2492–2502. (10.1016/j.neuroimage.2010.10.014) 20946964

[6] Rütgen M, Seidel EM, Silani G, Riečanský I, Hummer A, Windischberger C, Petrovic P, Lamm C. 2015 Placebo analgesia and its opioidergic regulation suggest that empathy for pain is grounded in self pain. Proc. Natl Acad. Sci. USA **112**, E5638–E5646. (10.1073/pnas.1511269112) 26417092 PMC4611649

[7] Heyes C. 2011 Automatic imitation. Psychol. Bull. **137**, 463–483. (10.1037/a0022288) 21280938

[8] Brass M, Heyes C. 2005 Imitation: is cognitive neuroscience solving the correspondence problem? Trends Cogn. Sci. **9**, 489–495. (10.1016/j.tics.2005.08.007) 16126449

[9] Decety J, Sommerville JA. 2003 Shared representations between self and other: a social cognitive neuroscience view. Trends Cogn. Sci. **7**, 527–533. (10.1016/j.tics.2003.10.004) 14643368

[10] Brass M, Ruby P, Spengler S. 2009 Inhibition of imitative behaviour and social cognition. Philos. Trans. R. Soc. Lond. B Biol. Sci. **364**, 2359–2367. (10.1098/rstb.2009.0066) 19620107 PMC2865080

[11] Quesque F, Brass M. 2019 The role of the temporoparietal junction in self-other distinction. Brain Topogr. **32**, 943–955. (10.1007/s10548-019-00737-5) 31676934

[12] de Vignemont F, Singer T. 2006 The empathic brain: how, when and why? Trends Cogn. Sci. **10**, 435–441. (10.1016/j.tics.2006.08.008) 16949331

[13] Darda KM, Ramsey R. 2019 The inhibition of automatic imitation: a meta-analysis and synthesis of fMRI studies. Neuroimage **197**, 320–329. (10.1016/j.neuroimage.2019.04.059) 31028924

[14] Bukowski H. 2018 The neural correlates of visual perspective taking: a critical review. Curr. Behav. Neurosci. Rep. **5**, 189–197. (10.1007/s40473-018-0157-6)

[15] Silani G, Lamm C, Ruff CC, Singer T. 2013 Right supramarginal gyrus is crucial to overcome emotional egocentricity bias in social judgments. J. Neurosci. **33**, 15466–15476. (10.1523/JNEUROSCI.1488-13.2013) 24068815 PMC6618458

[16] Eddy CM. 2016 The junction between self and other? Temporo-parietal dysfunction in neuropsychiatry. Neuropsychologia **89**, 465–477. (10.1016/j.neuropsychologia.2016.07.030) 27457686

[17] Bukowski H, Todorova B, Boch M, Silani G, Lamm C. 2021 Socio-cognitive training impacts emotional and perceptual self-salience but not self-other distinction. Acta Psychol. **216**, 103297. (10.1016/j.actpsy.2021.103297) 33773331

[18] de Guzman M, Bird G, Banissy MJ, Catmur C. 2016 Self-other control processes in social cognition: from imitation to empathy. Philos. Trans. R. Soc. Lond. B Biol. Sci. **371**, 20150079. (10.1098/rstb.2015.0079) 26644597 PMC4685524

[19] Qureshi AW, Monk RL, Samson D, Apperly IA. 2020 Does interference between self and other perspectives in theory of mind tasks reflect a common underlying process? Evidence from individual differences in theory of mind and inhibitory control. Psychon. Bull. Rev. **27**, 178–190. (10.3758/s13423-019-01656-z) 31429057 PMC7000534

[20] Santiesteban I, White S, Cook J, Gilbert SJ, Heyes C, Bird G. 2012 Training social cognition: from imitation to theory of mind. Cognition **122**, 228–235. (10.1016/j.cognition.2011.11.004) 22133627

[21] Tomova L, von Dawans B, Heinrichs M, Silani G, Lamm C. 2014 Is stress affecting our ability to tune into others? Evidence for gender differences in the effects of stress on self-other distinction. Psychoneuroendocrinology **43**, 95–104. (10.1016/j.psyneuen.2014.02.006) 24703175

[22] Tomova L, Heinrichs M, Lamm C. 2019 The other and me: effects of oxytocin on self-other distinction. Int. J. Psychophysiol. **136**, 49–53. (10.1016/j.ijpsycho.2018.03.008) 29550334

[23] Stroop JR. 1935 Studies of interference in serial verbal reactions. J. Exp. Psychol. **18**, 643–662. (10.1037/h0054651)

[24] Schurz M, Kronbichler M, Weissengruber S, Surtees A, Samson D, Perner J. 2015 Clarifying the role of theory of mind areas during visual perspective taking: issues of spontaneity and domain-specificity. Neuroimage **117**, 386–396. (10.1016/j.neuroimage.2015.04.031) 25907759

[25] Cole GG, Atkinson M, Le ATD, Smith DT. 2016 Do humans spontaneously take the perspective of others? Acta Psychol. **164**, 165–168. (10.1016/j.actpsy.2016.01.007) 26826864

[26] Conway JR, Lee D, Ojaghi M, Catmur C, Bird G. 2017 Submentalizing or mentalizing in a level 1 perspective-taking task: a cloak and goggles test. J. Exp. Psychol. Hum. Percept. Perform. **43**, 454–465. (10.1037/xhp0000319) 27893269 PMC5327864

[27] Furlanetto T, Becchio C, Samson D, Apperly I. 2016 Altercentric interference in level 1 visual perspective taking reflects the ascription of mental states, not submentalizing. J. Exp. Psychol. Hum. Percept. Perform. **42**, 158–163. (10.1037/xhp0000138) 26389611

[28] Marshall J, Gollwitzer A, Santos LR. 2018 Does altercentric interference rely on mentalizing? Results from two level-1 perspective-taking tasks. PLoS One **13**, e0194101. (10.1371/journal.pone.0194101) 29566019 PMC5864002

[29] Santiesteban I, Catmur C, Hopkins SC, Bird G, Heyes C. 2014 Avatars and arrows: implicit mentalizing or domain-general processing? J. Exp. Psychol. Hum. Percept. Perform. **40**, 929–937. (10.1037/a0035175) 24377486

[30] Santiesteban I, Kaur S, Bird G, Catmur C. 2017 Attentional processes, not implicit mentalizing, mediate performance in a perspective-taking task: evidence from stimulation of the temporoparietal junction. Neuroimage **155**, 305–311. (10.1016/j.neuroimage.2017.04.055) 28454821

[31] Vestner T, Balsys E, Over H, Cook R. 2022 The self-consistency effect seen on the dot perspective task is a product of domain-general attention cueing, not automatic perspective taking. Cognition **224**, 105056. (10.1016/j.cognition.2022.105056) 35149309

[32] Heyes C. 2014 Submentalizing: I am not really reading your mind. Perspect. Psychol. Sci. **9**, 131–143. (10.1177/1745691613518076) 26173251

[33] Quesque F, Rossetti Y. 2020 What do theory-of-mind tasks actually measure? Theory and practice. Perspect. Psychol. Sci. **15**, 384–396. (10.1177/1745691619896607) 32069168

[34] Holland C, Shin SM, Phillips J. Do you see what I see? A meta-analysis of the dot perspective task. https://escholarship.org/uc/item/7cs5r2xq.

[35] Bukowski H, Hietanen JK, Samson D. 2015 From gaze cueing to perspective taking: revisiting the claim that we automatically compute where or what other people are looking at. Vis. cogn. **23**, 1020–1042. (10.1080/13506285.2015.1132804) 26924936 PMC4743615

[36] Bukowski H, Samson D. 2021 Automatic imitation is reduced in narcissists but only in egocentric perspective-takers. Acta Psychol. **213**, 103235. (10.1016/j.actpsy.2020.103235) 33321398

[37] Samson D, Apperly IA. 2010 There is more to mind reading than having theory of mind concepts: new directions in theory of mind research. Infant Child Dev. **19**, 443–454. (10.1002/icd.678)

[38] Ramsey R. 2018 What are reaction time indices of automatic imitation measuring? Conscious. Cogn. **65**, 240–254. (10.1016/j.concog.2018.08.006) 30219745

[39] Shaw DJ, Czekóová K, Porubanová M. 2017 Orthogonal-compatibility effects confound automatic imitation: implications for measuring self-other distinction. Psychol. Res. **81**, 1152–1165. (10.1007/s00426-016-0814-x) 27752773

[40] Cooper RP, Catmur C, Heyes C. 2013 Are automatic imitation and spatial compatibility mediated by different processes? Cogn. Sci. **37**, 605–630. (10.1111/j.1551-6709.2012.01252.x) 22578089

[41] Cracco E, Bardi L, Desmet C, Genschow O, Rigoni D, De Coster L, Radkova I, Deschrijver E, Brass M. 2018 Automatic imitation: a meta-analysis. Psychol. Bull. **144**, 453–500. (10.1037/bul0000143) 29517262

[42] Bukowski H, Lamm C. 2020 Temporoparietal junction. In Encyclopedia of personality and individual differences, pp. 5413–5417. Cham, Switzerland: Springer. (10.1007/978-3-319-24612-3_863)

[43] Schuwerk T, Schurz M, Müller F, Rupprecht R, Sommer M. 2017 The rTPJ’s overarching cognitive function in networks for attention and theory of mind. Soc. Cogn. Affect. Neurosci. **12**, 157–168. (10.1093/scan/nsw163) 27798260 PMC5390694

[44] Happé F, Cook JL, Bird G. 2017 The structure of social cognition: in(ter)dependence of sociocognitive processes. Annu. Rev. Psychol. **68**, 243–267. (10.1146/annurev-psych-010416-044046) 27687121

[45] Sowden S, Catmur C. 2015 The role of the right temporoparietal junction in the control of imitation. Cereb. Cortex **25**, 1107–1113. (10.1093/cercor/bht306) 24177989 PMC4380005

[46] Samson D, Apperly IA, Braithwaite JJ, Andrews BJ, Bodley Scott SE. Seeing it their way: Evidence for rapid and involuntary computation of what other people see. J. Exp. Psychol. **36**, 1255–1266. (10.1037/a0018729) 20731512

[47] von Mohr M, Finotti G, Ambroziak KB, Tsakiris M. 2020 Do you hear what i see? An audio-visual paradigm to assess emotional egocentricity bias. Cog. Emot. **34**, 756–770. (10.1080/02699931.2019.1683516) 31672095

[48] Logan GD, Cowan WB. 1984 On the ability to inhibit thought and action: a theory of an act of control. Psychol. Rev. **91**, 295–327. (10.1037/0033-295X.91.3.295) 24490789

[49] Verbruggen F *et al*. 2019 A consensus guide to capturing the ability to inhibit actions and impulsive behaviors in the stop-signal task. eLife **8**, e46323. (10.7554/eLife.46323) 31033438 PMC6533084

[50] Bock O, Baetge I, Nicklisch A. 2014 Hroot: hamburg registration and organization online tool. Eur. Econ. Rev. **71**, 117–120. (10.1016/j.euroecorev.2014.07.003)

[51] Oldfield RC. 1971 The assessment and analysis of handedness: the Edinburgh inventory. Neuropsychologia **9**, 97–113. (10.1016/0028-3932(71)90067-4) 5146491

[52] Brass M, Derrfuss J, von Cramon DY. 2005 The inhibition of imitative and overlearned responses: a functional double dissociation. Neuropsychologia **43**, 89–98. (10.1016/j.neuropsychologia.2004.06.018) 15488909

[53] Parris BA, Sharma D, Weekes BSH, Momenian M, Augustinova M, Ferrand L. 2019 Response modality and the stroop task: are there phonological stroop effects with manual responses? Exp. Psychol. **66**, 361–367. (10.1027/1618-3169/a000459) 31696793

[54] Simon JR, Sudalaimuthu P. 1979 Effects of s mapping and response modality on performance in a stroop task. J. Exp. Psychol. **5**, 176–187. (10.1037//0096-1523.5.1.176) 528927

[55] Wood JN. 2008 Visual memory for agents and their actions. Cognition **108**, 522–532. (10.1016/j.cognition.2008.02.012) 18472092

[56] Cooper RP, Byde C, de Cecilio R, Fulks C, Morais DS. 2018 Set-shifting and place-keeping as separable control processes. Cogn. Psychol. **105**, 53–80. (10.1016/j.cogpsych.2018.07.001) 30032062

[57] Pronizius E, Bukowski H, Lamm C. 2024 Data from: Comparing self-other distinction across motor, cognitive, and affective domains. Figshare. (10.6084/m9.figshare.c.7452089) PMC11461050 39386983

[58] Sowden S, Koehne S, Catmur C, Dziobek I, Bird G. 2016 Intact automatic imitation and typical spatial compatibility in autism spectrum disorder: challenging the broken mirror theory. Autism Res. **9**, 292–300. (10.1002/aur.1511) 26112060

[59] Samson D, Apperly I. 2015 Level 1 Visual Perspective-Taking Task. figshare. Dataset. (10.6084/m9.figshare.1455943)

[60] Liesefeld HR, Janczyk M. 2019 Combining speed and accuracy to control for speed-accuracy trade-offs(?). Behav. Res. Methods **51**, 40–60. (10.3758/s13428-018-1076-x) 30022459

[61] Leontyev A. 2021 SSRTcalc: Easy SSRT calculation [Internet]. See https://CRAN.R-project.org/package=SSRTcalc.

[62] Schönbrodt FD, Perugini M. 2013 At what sample size do correlations stabilize? J. Res. Pers. **47**, 609–612. (10.1016/j.jrp.2013.05.009)

[63] Schönbrodt FD, Perugini M. 2018 Corrigendum to “at what sample size do correlations stabilize?” J. Res. Pers. **74**, 609–612. (10.1016/j.jrp.2018.02.010)

[64] Shaw DJ, Czekóová K, Pennington CR, Qureshi AW, Špiláková B, Salazar M, Brázdil M, Urbánek T. 2020 You ≠ me: individual differences in the structure of social cognition. Psychol. Res. **84**, 1139–1156. (10.1007/s00426-018-1107-3) 30324265 PMC7239802

[65] Craft JL, Simon JR. 1970 Processing symbolic information from a visual display: interference from an irrelevant directional cue. J. Exp. Psychol. **83**, 415–420. (10.1037/h0028843) 4098174

[66] Halkidi M. 2009 Hierarchical clustering. In Encyclopedia of database systems. Boston, MA: Springer. (10.1007/978-0-387-39940-9_604)

[67] R Core Team. 2023 R: A language and environment for statistical computing. Vienna, Austria: R Foundation for Statistical Computing. See https://www.R-project.org/.

[68] Davison ML, Sireci SG. 2000 Multidimensional scaling. in: handbook of applied multivariate statistics and mathematical modeling, pp. 323–352. Academic Press.

[69] Carver CS, White TL. 1994 Behavioral inhibition, behavioral activation, and affective responses to impending reward and punishment: the BIS/BAS scales. J. Pers. Soc. Psychol. **67**, 319–333. (10.1037/0022-3514.67.2.319)

[70] Watson D, Clark LA, Tellegen A. 1988 Development and validation of brief measures of positive and negative affect: the PANAS scales. J. Pers. Soc. Psychol. **54**, 1063–1070. (10.1037/0022-3514.54.6.1063) 3397865

[71] Kroenke K, Spitzer RL, Williams JB. 2001 The PHQ-9: validity of a brief depression severity measure. J. Gen. Intern. Med. **16**, 606–613. (10.1046/j.1525-1497.2001.016009606.x) 11556941 PMC1495268

[72] Spitzer RL, Kroenke K, Williams JBW, Löwe B. 2006 A brief measure for assessing generalized anxiety disorder. Arch. Intern. Med. **166**, 1092. (10.1001/archinte.166.10.1092) 16717171

[73] Reniers RL, Corcoran R, Drake R, Shryane NM, Völlm BA. 2011 The QCAE: a questionnaire of cognitive and affective empathy. J. Pers. Assess. **93**, 84–95. (10.1080/00223891.2010.528484) 21184334

[74] Bagby RM, Parker JD, Taylor GJ. 1994 The twenty-item toronto alexithymia scale-i. item selection and cross-validation of the factor structure. J. Psychosom. Res. **38**, 23–32. (10.1016/0022-3999(94)90005-1) 8126686

[75] Russell DW. 1996 UCLA loneliness scale (version 3): reliability, validity, and factor structure. J. Pers. Assess. **66**, 20–40. (10.1207/s15327752jpa6601_2) 8576833

[76] Loenneker HD *et al*. 2024 We don’t know what you did last summer. On the importance of transparent reporting of reaction time data pre-processing. Cor. **172**, 14–37. (10.1016/j.cortex.2023.11.012) 38154375

[77] Stanley DJ. 2021 apaTables: Create American psychological association (APA) style tables [Internet]. See https://CRAN.R-project.org/package=apaTables.

[78] Stanley DJ, Spence JR. 2018 Reproducible tables in psychology using the apatables package. Adv. Methods Pract. Psychol. Sci. **1**, 415–431. (10.1177/2515245918773743)

[79] Galili T. 2015 Dendextend: an R package for visualizing, adjusting and comparing trees of hierarchical clustering. Bioinformatics **31**, 3718–3720. (10.1093/bioinformatics/btv428) 26209431 PMC4817050

[80] Wickham H, François R, Henry L, Müller K, Vaughan D. 2023 Dplyr: a grammar of data manipulation [Internet]. See https://CRAN.R-project.org/package=dplyr.

[81] Ben-Shachar M, Lüdecke D, Makowski D. 2020 Effectsize: estimation of effect size indices and standardized parameters. J. Open Source Software **5**, 2815. (10.21105/joss.02815)

[82] Lenth RV. 2024 Emmeans: estimated marginal means, aka least-squares means [Internet]. See https://CRAN.R-project.org/package=emmeans.

[83] Gohel D, Skintzos P. 2023 Flextable: functions for tabular reporting [Internet]. See https://CRAN.R-project.org/package=flextable.

[84] Wickham H. 2016 Ggplot2: Elegant graphics for data analysis [Internet]. Springer-Verlag New York. See https://ggplot2.tidyverse.org.

[85] Kassambara A. 2023 Ggpubr: ’ggplot2’ based publication ready plots [Internet]. See https://CRAN.R-project.org/package=ggpubr.

[86] Müller K. 2020 Here: a simpler way to find your files [Internet]. See https://CRAN.R-project.org/package=here.

[87] Long JA. 2019 Interactions: comprehensive, user-friendly toolkit for probing interactions [Internet]. See https://cran.r-project.org/package=interactions.

[88] Long JA. 2022 Jtools: analysis and presentation of social scientific data [Internet]. See https://cran.r-project.org/package=jtools.

[89] Kuznetsova A, Brockhoff PB, Christensen RHB. 2017 Lmertest package: tests in linear mixed effects models. J. Stat. Softw. **82**, 1–26. (10.18637/jss.v082.i13)

[90] Bloggs J. 2014 Mergeutils: functions to aid merging and checking disparate data [Internet]. See https://rdrr.io/github/vapniks/mergeutils/.

[91] Gohel D. 2023 Officer: manipulation of microsoft word and powerpoint documents [Internet]. See https://CRAN.R-project.org/package=officer.

[92] Chan C, Leeper TJ, Becker J, Schoch D. 2023 Rio: a swiss-army knife for data file i/o [Internet]. See https://cran.r-project.org/package=rio.

[93] Hope RM. 2022 Rmisc: ryan miscellaneous [Internet]. See https://CRAN.R-project.org/package=Rmisc.

[94] Lüdecke D. 2023 sjPlot: Data visualization for statistics in social science [Internet]. See https://CRAN.R-project.org/package=sjPlot.

[95] Müller K, Wickham H. 2023 Tibble: Simple data frames [Internet]. See https://CRAN.R-project.org/package=tibble.

[96] Wickham H, Vaughan D, Girlich M. 2024 Tidyr: tidy messy data [Internet]. See https://CRAN.R-project.org/package=tidyr.

[97] Wickham H *et al*. 2019 Welcome to the tidyverse. J. Open Source Software **4**, 1686. (10.21105/joss.01686)

[98] Ooms J. Writexl: export data frames to excel ’xlsx format [Internet]. See https://CRAN.R-project.org/package=writexl.

[99] Martin AK, Perceval G, Davies I, Su P, Huang J, Meinzer M. 2019 Visual perspective taking in young and older adults. J. Exp. Psychol. **148**, 2006–2026. (10.1037/xge0000584) 30985182

[100] May M, Wendt M. 2013 Visual perspective taking and laterality decisions: problems and possible solutions. Front. Hum. Neurosci. **7**, 549. (10.3389/fnhum.2013.00549) 24046744 PMC3764372

[101] Khng KH, Lee K. 2014 The relationship between stroop and stop-signal measures of inhibition in adolescents: influences from variations in context and measure estimation. PLoS One **9**, e101356. (10.1371/journal.pone.0101356) 24992683 PMC4081588

[102] Hedge C, Powell G, Sumner P. 2018 The reliability paradox: why robust cognitive tasks do not produce reliable individual differences. Behav. Res. Methods. **50**, 1166–1186. (10.3758/s13428-017-0935-1) 28726177 PMC5990556

[103] Draheim C, Mashburn CA, Martin JD, Engle RW. 2019 Reaction time in differential and developmental research: a review and commentary on the problems and alternatives. Psychol. Bull. **145**, 508–535. (10.1037/bul0000192) 30896187

[104] Bukowski H, Tik M, Silani G, Ruff CC, Windischberger C, Lamm C. 2020 When differences matter: rTMS/fMRI reveals how differences in dispositional empathy translate to distinct neural underpinnings of self-other distinction in empathy. Cortex. **128**, 143–161. (10.1016/j.cortex.2020.03.009) 32335328

[105] Bukowski H, Samson D. 2017 New insights into the inter-individual variability in perspective taking. Vision. **1**, 1–8. (10.3390/vision1010008) 31740633 PMC6835961

[106] Samuel S, Cole GG, Eacott MJ. 2023 It’s not you, it’s me: a review of individual differences in visuospatial perspective taking. Perspect. Psychol. Sci. **18**, 293–308. (10.1177/17456916221094545) 35994772 PMC10018059

[107] Genschow O, van Den Bossche S, Cracco E, Bardi L, Rigoni D, Brass M. 2017 Mimicry and automatic imitation are not correlated. PLoS One **12**, e0183784. (10.1371/journal.pone.0183784) 28877197 PMC5587324

[108] von Mohr M, Finotti G, Villani V, Tsakiris M. 2021 Taking the pulse of social cognition: cardiac afferent activity and interoceptive accuracy modulate emotional egocentricity bias. Cortex **145**, 327–340. (10.1016/j.cortex.2021.10.004) 34794068

[109] Goregliad Fjaellingsdal T, Makowka N, Krämer UM. 2023 Studying trait-characteristics and neural correlates of the emotional ego- and altercentric bias using an audiovisual paradigm. Cogn. Emot. **37**, 818–834. (10.1080/02699931.2023.2211253) 37203227

[110] Kanske P, Böckler A, Trautwein FM, Singer T. 2015 Dissecting the social brain: introducing the empatom to reveal distinct neural networks and brain-behavior relations for empathy and theory of mind. Neuroimage **122**, 6–19. (10.1016/j.neuroimage.2015.07.082) 26254589

[111] Depow GJ, Francis Z, Inzlicht M. 2021 The experience of empathy in everyday life. Psychol. Sci. **32**, 1198–1213. (10.1177/0956797621995202) 34241543 PMC13038167

[112] Simons DJ, Shoda Y, Lindsay DS. 2017 Constraints on generality (COG): a proposed addition to all empirical papers. Perspect. Psychol. Sci. **12**, 1123–1128. (10.1177/1745691617708630) 28853993

[113] Olsson-Collentine A, van Aert R, Bakker M, Wicherts J. 2023 Meta-analyzing the multiverse: a peek under the hood of selective reporting. Psychol. Methods. (10.1037/met0000559) 37166859

[114] Steegen S, Tuerlinckx F, Gelman A, Vanpaemel W. 2016 Increasing transparency through a multiverse analysis. Perspect. Psychol. Sci. **11**, 702–712. (10.1177/1745691616658637) 27694465

[115] Höfler M, Scherbaum S, Kanske P, McDonald B, Miller R. 2022 Means to valuable exploration: I. the blending of confirmation and exploration and how to resolve it. Meta Psychology. **6**. (10.15626/MP.2021.2837)

[116] Höfler M, McDonald B, Kanske P, Miller R. 2023 Means to valuable exploration II: how to explore data to modify existing claims and create new ones. Meta Psychology **7**. (10.15626/MP.2022.3270)

[117] Eddy CM. 2018 Social cognition and self-other distinctions in neuropsychiatry: insights from schizophrenia and tourette syndrome. Prog. Neuropsychopharmacol. Biol. Psychiatry. **82**, 69–85. (10.1016/j.pnpbp.2017.11.026) 29195921

[118] Simonsen A, Fusaroli R, Skewes JC, Roepstorff A, Campbell-Meiklejohn D, Mors O, Bliksted V. 2019 Enhanced automatic action imitation and intact imitation-inhibition in schizophrenia. Schizophr. Bull. **45**, 87–95. (10.1093/schbul/sby006) 29474687 PMC6293210

[119] Rudolph A, Liepelt R, Kaffes M, Hofmann-Shen C, Montag C, Neuhaus AH. 2022 Motor cognition in schizophrenia: control of automatic imitation and mapping of action context are reduced. Schizophr. Res. **240**, 116–124. (10.1016/j.schres.2021.12.024) 34995996

[120] De Meulemeester C, Lowyck B, Luyten P. 2021 The role of impairments in self–other distinction in borderline personality disorder: a narrative review of recent evidence. Neurosci. Biobehav. Rev. **127**, 242–254. (10.1016/j.neubiorev.2021.04.022) 33901500

[121] Pronizius E, Bukowski H, Lamm C. 2024 Comparing Self-Other Distinction Across Motor, Cognitive, and Affective Domains. See 10.17605/OSF.IO/NYS7Q. PMC11461050 39386983

[122] Pronizius E, Bukowski H, Lamm C. 2022 Comparing Self-Other Distinction Across Motor, Cognitive, and Affective Domains. See 10.31234/osf.io/b2pv9. PMC11461050 39386983

